# Transferability of a US claims-based machine learning model for ATTRwt-CM identification: a retrospective evaluation in a German setting

**DOI:** 10.1038/s41598-026-49636-3

**Published:** 2026-04-24

**Authors:** Harisa Muratovic-Colic, Miriam R. Hübner, Jan-Filip Rehburg, Isabel Mattig, Paul J. Wetzel, Katrin Wrede-Wihl, Helena F. Pernice, Gina Barzen, Nicolas Wieder, Jakub Piwowarski, Stephan Bohl, Vera von Landenberg-Roberg, Daniel Messroghli, Sebastian Spethmann, Richard Röttger, Josef Schepers, Katrin Hahn

**Affiliations:** 1 Amyloidosis Center Charité Berlin (ACCB), Charité Universitätsmedizin Berlin, Berlin, Germany; 2 Klinik für Neurologie mit Experimenteller Neurologie, Charité Universitätsmedizin Berlin, Berlin, Germany; 3 Berlin Institute of Health (BIH) at Charité, Charité Universitätsmedizin Berlin, Berlin, Germany; 4 Charité – Universitätsmedizin Berlin, corporate member of Freie Universität Berlin and Humboldt-Universität zu Berlin, Charitéplatz 1, 10117 Berlin, Germany; 5 DZHK (German Centre for Cardiovascular Research), Partner Site Berlin, Berlin, Germany; 6 Charité Universitätsmedizin Berlin, Berlin, Germany; 7 Department of Cardiology, Angiology and Intensive Care Medicine, Campus Charité Mitte, Deutsches Herzzentrum der Charité (DHZC), Charitéplatz 1, 10117 Berlin, Germany; 8 Department of Cardiology, Rhythmology and Angiology, Medical University of Lausitz - Carl Thiem, Cottbus, Germany; 9 Medizinische Klinik mit Schwerpunkt Hämatologie, Onkologie und Tumorimmunologie, Charité Universitätsmedizin Berlin, Berlin, Germany; 10 University of Southern Denmark, Department of Mathematics and Computer Science, Odense, Denmark

**Keywords:** Heart failure, Cardiomyopathy, Systemic disease, Machine learning, Rare diseases, Cardiology, Computational biology and bioinformatics, Diseases, Medical research

## Abstract

Cardiac amyloidosis (CA), a fatal and progressive cardiomyopathy is characterized by amyloid deposition within the myocardium. The main forms of CA include transthyretin amyloidosis (ATTR-CM; distinguished into a hereditary and wildtype form [ATTRv and ATTRwt]) and light-chain amyloidosis (AL). Transthyretin amyloidosis with cardiomyopathy (ATTR-CM) is increasingly recognized among heart failure (HF) patients, despite its underestimated prevalence as a rare disease. ATTR-CM is challenging to diagnose due to its broad phenotypic overlap with HF, yet a timely differentiation of CA is crucial, as effective treatment can significantly improve quality of life. Machine learning (ML) approaches could enable earlier detection of ATTR-CM in routine care data. Data of ATTR-CM patients from the Amyloidosis Center Charité Berlin (2002-2023) and data from a cohort with heart failure and aortic stenosis screened for ATTR-CM were mapped from ICD-10-GM to ICD-10-CM and evaluated with an open-source random forest (RF) model based on ICD-10-CM codes which was previously validated in multiple external cohorts in the US and UK. In the cohort of patients with HF and aortic stenosis, cases of ATTR-CM were recognized using the algorithm, although it was not possible to distinguish between ATTRv-CM and ATTRwt-CM. Sensitivity and specificity were significantly lower than in the external validations in the US and UK. Model performance depended strongly on coding granularity and feature availability and contributed to the diminished predictive power. While the RF model showed moderate transferability under enriched conditions, reliance of geographically specific ICD-10 systems limits broader applicability due to coding discrepancies and information loss. These findings underscore the need for improved semantic harmonization of routine data, as well as the integration of rare disease specific ontologies such as the Human Phenotype Ontology (HPO) and ORPHAcodes to enhance cross-border ML-based assistance for timely diagnosing patients with rare diseases.

## Introduction

Cardiac amyloidosis (CA), a form of fatal and progressive cardiomyopathy, encapsulates conditions characterized by deposition of misfolded protein aggregates within the myocardium. The main forms of CA include transthyretin amyloidosis (ATTR) and light-chain amyloidosis (AL). AL is caused by the deposition of immunoglobulin light chains fragments, typically due to plasma cell dyscrasias like multiple myeloma, and is presenting as the phenocopy of hypertrophic and restrictive cardiomyopathies. ATTR is further divided into two categories: (i) wild type transthyretin amyloidosis, with a predominant cardiological manifestation (ATTRwt-CM), and (ii) variant transthyretin amyloidosis (ATTRv), well known to compromise heart and the nervous system, generally in the form of polyneuropathies. ATTR-CM occurs under both conditions, although distinguishing between ATTRwt-CM and ATTRv-CM may be clinically relevant, this distinction is not central to the objectives of this work.

ATTRwt-CM, previously known as senile systemic amyloidosis, is an age-related, idiopathic disease affecting mostly male patients older than 60 years. The prevalence has been estimated to 1/5800^1^ patients worldwide and is thereby considered a rare disease. The median survival of patients without treatment is 47 months, with 78% of deaths occurring due to cardiac-related events^2^. With a wide range of causal treatment options available, the responsibility to diagnose patients with ATTR-CM and refer them for treatment has increased further. However, the diagnosis of ATTR-CM is challenging due to the non-specific symptoms such as shortness of breath, cardiac arrhythmia, chest pain, oedema, fatigue, or exercise intolerance. As these symptoms occur frequently in various heart diseases, ATTR-CM may be overlooked. Other non-cardiac ATTR amyloidosis symptoms include carpal tunnel syndrome, lumbar spinal stenosis, distal biceps tendon rupture as well as sensory polyneuropathy^3,4^. In addition, multiple studies demonstrated a higher prevalence of aortic stenosis in ATTR-CM which can complicate diagnosis and management^5,6^. ATTRwt-CM was previously considered as predominantly associated with HF with preserved ejection fraction (HFpEF)^7^. However, it seems that patients with HF with reduced ejection fraction (HFrEF) might also be at considerable risk of exhibiting ATTR-CM, which challenges the disease’s association with only HFpEF^8^. Effective disease-modifying treatments for both forms of CA exist – AL is successfully treated with chemotherapy^9^, and ATTR-CM specifically with tafamidis^10^, vutrisiran^11^ or acoramidis^12^. Considering the vulnerable target population, the radically reduced quality of life associated with the disease, and the availability of functional inhibitors that can mitigate amyloid deposition, it becomes pivotal to join hands in exploring methods for the timely diagnosis of affected patients.

Leveraging the power of machine learning to support clinical decision-making holds significant potential in improving diagnostic accuracy and patient care. Huda et al^13^. created a promising ML algorithm for identifying patients at risk for developing ATTRwt-CM in 2021. Their algorithm was trained on medical claims data (data collected from bills, doctor’s appointments, insurance information and other patient-provider information) using the U.S.-specific ICD-10-CM (International Classification of Diseases, 10th revision - Clinical Modification) coding system. It was validated across four different cohorts in the US and UK. However, the UK-based data had to be subjected to a mapping of local coding systems (ICD-10-WHO and Read codes) to the ICD-10-CM framework on which the algorithm is based^14^, already imposing limitations. Despite achieving appropriate sensitivity and specificity on the balanced IQVIA^18^ dataset, validating the RF model on a much larger, unbalanced data set (Northwestern Medical Enterprise Data Warehouse), the number of false positives exceeded the number of true positives significantly. This observation is consistent with Bayes’ theorem^15^. In low prevalence diseases, even a model with a good sensitivity and specificity would yield a low positive predictive value (PPV), due to the small prior probability of the disease. Consequently, the proportion of true positive cases is decreased, while false positives accumulate simply due to the much larger number of non-diseased individuals. Therefore, we face an additional limitation – detecting ATTR-CM, accompanied by the burden of high false positives when screening larger populations.

An evaluation of Huda et al.’s^13^ random forest (RF) model to detect ATTR-CM in patients with HF and aortic stenosis is presented here, focusing both on its underlying ML structure and the consequences of utilizing geographically specific coding systems for patient identification in various scenarios, especially when using semantically non-harmonized medical data.

Inspired by the validation of the RF model in a UK setting, we set our main objectives to address the ICD-10-CM as the basis of a prediction model for patients at risk for a rare disease and the corresponding state of a single center regarding ICD-10-GM coded data, as well as its eligibility for RF model validation. Our contribution focuses on critically evaluating the transportability of ML as a potential screening instrument for identifying at-risk patients in a constrained, single-center setting, and examining under which conditions its implementation in real-world clinical workflows is currently achievable. We further evaluated the transferability of algorithms trained on American data to European, more specifically German data. Since different coding systems are used, information loss and therefore classification performance drops are expected, and are quantified within the single center German setting. Finally, we address the feasibility of future steps with respect to the expansion of data originating in the data integration centers (DIC) of German university clinics that are harmonized based on multi-institutional semantic agreements to serve as a grid for identifying symptom relations from a unified, nationally representative database. Important considerations are also given to the fact, that in population wide screening efforts, we are dealing with highly skewed class proportions in the case of rare diseases, directly affecting the PPV as a screening tool.

## Results

### Random forest model background

**Fig. 1:**
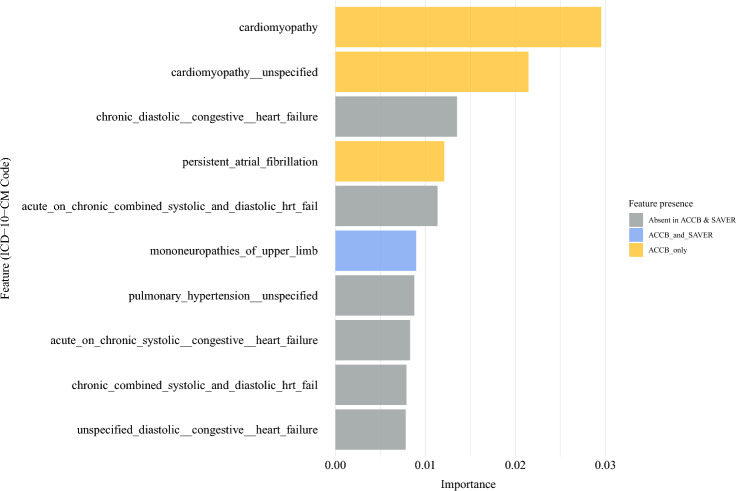
The top ten important features for the model presented by Huda et al^13^. are shown, color coded with respect to the presence in the German cohort, SAVER and ACCB. The features in yellow are captured within the ACCB only (relating to HF), whereas the one presented in blue (mononeuropathies of the upper limbs) indicates a presence in both ACCB and SAVER. Grey colored features are absent from the GM system underscoring the granularity ambiguities of disease coding in CM and GM.

The RF model evaluated in this section is presented in the study conducted by Huda et al^13^. It was trained on the propensity-matched dataset curated from IQVIA Inc. (Durham, NC)^18^, which included 1071 positive cases of ATTRwt-CM and 1071 negative cases (i.e. cardiomyopathy/HF without ATTRwt) The RF model achieved a sensitivity, specificity, and accuracy of 87% each, a PPV of 88% and an NPV of 86% and finally, the AUROC of 97% in the US setting. Noteworthy, the high PPV is owed to a balanced input size with equal number of patients for the negative and positive class. Nevertheless, such balance does not reflect the real-world settings when screening large populations for the identification of individuals affected by a rare disease. In such cases, we deal with the problem of many false positives which would significantly decrease the PPV, since the non-disease group vastly outnumbers the disease group in the population. Furthermore, Huda et al^13^. have also ranked the most important features of the RF model (i.e. patient diagnoses) according to the model’s innate feature importance on their training dataset, with the goal of gaining knowledge about which features were the best indicators for patients at risk for ATTRwt-CM. Their 10 most important features for the model’s predictions, depicted in Fig. 1, predominantly correspond to various forms of cardiomyopathies, HF, and neuropathic afflictions. It is important to note that six of the top ten features, namely chronic diastolic congestive HF, acute on chronic systolic/combined systolic and diastolic HF, chronic combined systolic and diastolic HF, and unspecified diastolic congestive HF are absent from the ICD-10-GM coding system. This discrepancy between the GM and CM alone was expected to hinder the model’s performance on the German dataset.

### ICD-10-GM to ICD-10-CM mapping results

**Fig. 2:**
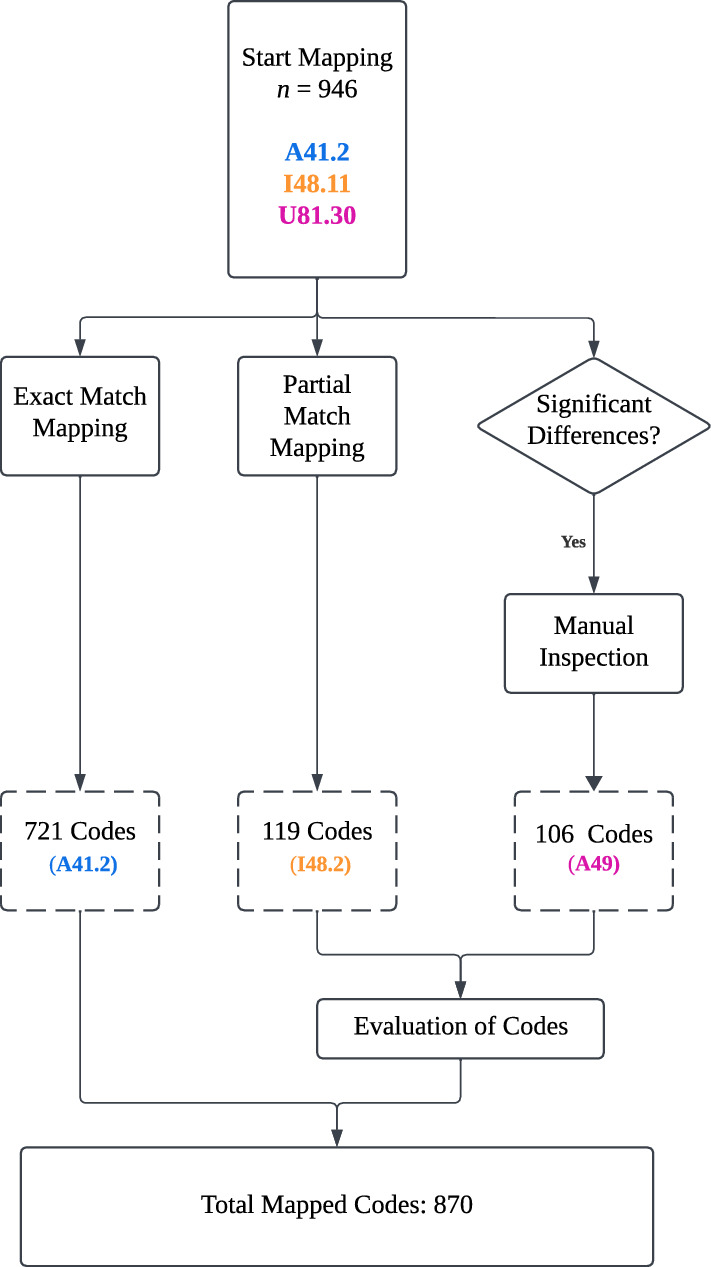
Examples of ICD-10-GM to ICD-10-CM code mapping approach with varying degrees of equivalence. Exact match mapping: A41.2 (blue) “Sepsis due to unspecified staphylococcus” is a 1:1 match based on code and definition in both systems. Partial match mapping: GM I48.11 (orange) “Chronic atrial fibrillation” maps to CM I48.2 with the same clinical meaning. Codes falling outside the scope of the exact and partial mapping are characterized by significant differences across their entire sequence, thereby reflecting the necessity to search for equivalents in distinct chapters. This is depicted in manual inspection mapping: GM U81.30 (magenta) “Pseudomonas aeruginosa with multidrug resistance 3MRGN” maps to the broader CM A49 “Bacterial infection of unspecified site”, illustrating loss of definition specificity. The subsets of partial and manual inspection were evaluated by physicians. As a result, the 946 initial GM codes were mapped to 870 CM codes.

The ICD-10 mapping from GM to CM for the ACCB patient group was conducted in a modular manner given the discrepancies between the coding systems. Exact match mapping, which required complete alignment of the entire code sequence between the GM and CM, yielded 721 matches. Partial mapping, conducted using the first 3 symbols of the code sequence, generated additional 119 matches. For the remaining 106 codes that showed substantial divergence between coding systems, a manual inspection was performed to determine and assign their appropriate definitions. For the data from ACCB, the GM to CM mapping resulted in 870 ICD-10-CM codes. The difference between the initial 946 and the resulting 870 mapped codes is a result of the mapping nature in which partially mismatched codes were mapped to a higher hierarchy (i.e. closest relative). An overview of the modular mapping can be seen in Fig. 2.

**Fig. 3:**
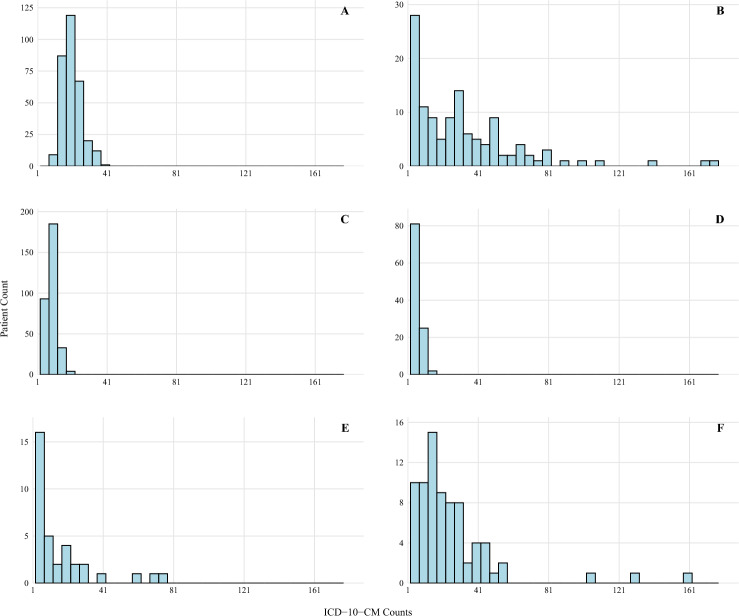
Distribution of hierarchically mapped ICD-10-CM codes included in the RF model for the diagnosis codes in the German scenarios (read row-wise). The first row covering A and B corresponds to the intact features SAVER and ACCB (A, left: SAVER patients’ mapped questionnaire-based diagnoses; B, right: ACCB’s patients’ longitudinal clinical record (2002-2023)). In A, a roughly bell-shaped distribution is centered around 25 ICD-10-CM codes per patient. In B, a slightly right-skewed distribution of codes and a long tail extending toward higher counts is shown. The feature constrained S1 is showcased in C and D and appropriately reflects similar profiles for allowed features for ACCB and SAVER. Finally, a representation of the number of features in S2 originating both in ACCB: left, E for the HF group’s feature counts and on the right, F, the positive class’ feature counts.

The ICD-10 mapping of SAVER’s diagnoses was treated differently due to its nature as a data set based on questionnaires. From these, appropriate matches between the diagnosis questions and the diagnoses captured in the CM system were directly mapped, and resulted in a total of 49 unique ICD-10-CM codes. An algorithm of the mapping approach is provided in the Supplementary Methods. To gain knowledge about the differences between the ACCB and SAVER cohort across the scenarios with respect to their origin and nature, a histogram of mapped codes for the respective cohorts can be consulted in Fig. 3. A heterogeneity of the retrospectively gathered ICD-10 information in a time frame 2002-2023 can be noted by the right-skewed distribution for the ACCB cohort in Fig. 3 on the right-hand side (3B). Such heterogeneity is expected, since the ACCB is a retrospective cohort collected over time, in which we can observe different individual temporal disease trajectories. On the left-hand side (3A), a roughly bell-shaped distribution of the SAVER patients’ diagnoses can be observed and is a result of all subjects being presented with the same questionnaires. Important to note in the first row is the highly differing diagnosis count for ACCB and SAVER, revealing the necessities for the definition of the two scenarios (S1 and S2). In the second row of Fig. 3, the uniform pattern seen in both C and D is a result of the feature constraint imposed on the ACCB group. The last row, which represents the diagnoses counts for the data originating in ACCB shows an increase of number of diagnoses for the negative (HF) and positive class (E85.4) presented to the RF model.

**Fig. 4:**
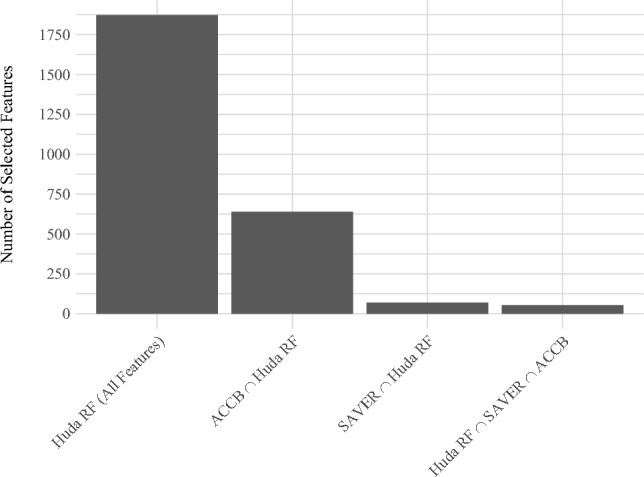
An overview of the total number of features relative to the features available in the two German groups (ACCB and SAVER). The RF contains 1872 features. 640 of them are shared with the ACCB, whereas only 70 with SAVER. The intersection between the total number of the RF features, SAVER and ACCB is 54.

Prior to the step of validating the RF model on the German cohort, we employed the hierarchical mapping provided in the supplementary data of Huda et al^13^. With this, the ICD-10-CM codes are hierarchically mapped to ensure a systematic strategy of obtaining a comprehensive diagnostic code classification. This mapping corresponds to a mapping within the CM system and is not to be mistaken with the GM to CM mapping. It is established by using the subchapter (diagnosis category), major (diagnosis name), and short description (diagnosis description) levels, utilizing the 2016 release of ICD-10-CM and updated for new codes introduced in the 2019 release. The feature was marked as present for the RF model, if the patients’ diagnosis code fell within the scope of any of the three levels (category, name and description). Such a methodological design provided multiple classificatory opportunities, thereby minimizing the risk of excluding potentially relevant diagnostic information. By progressively expanding the mapping criteria from the most granular level available to increasingly broader diagnostic categories (i.e. levels), Huda et al^13^. maximized diagnostic code inclusivity while maintaining a structured and reproducible mapping process. For example, a patient assigned the diagnosis code corresponding to “heart failure, unspecified” would be encoded as positive for that specific feature as well as for its parent category, namely “heart failure”. As a result of this hierarchical mapping process, of the 1,872 features in the model, the German validation cohort contained 34% of the total feature set in the RF model (p=640). An overview of the intersections with the RF feature set and the German cohort (SAVER, ACCB) is depicted in Fig. 4.

### Performance evaluation

Comparing the performance of Huda et al.’s^13^ RF model on the German cohort with respect to the two scenarios showcases distinct differences, both indicating that its performance remains insufficient for clinical use. A direct comparison to the closest, US-based scenario (NMEDW) can be consulted due to the same target variable used (E85.4) as well as the class imbalance in S2.

#### Scenario I

Sensitivity was low across all subscenarios (11.47–18.42.47.42%), indicating very low ability to recall the positive cases and is likely attributed to the reduced number of allowed features. Large specificity scores were found among the three scenarios (82.08–83.28.08.28%) showcasing a good ability to identify negative cases, despite the class imbalance in S1. Accuracy ranging from 67.35% to 72.18% is most likely driven by the predominance of the negative cases, whereas the AUROC values show a no better prediction than at random (46.98–51.48.98.48%). Since the data is characterized by a class imbalance, the low F1-score is a result of low sensitivity and PPV. The Matthews correlation coefficient (MCC), indicative of how well the model was able to predict the positive class ranged from −0.05 to 0.01 suggesting very little predictive correlation. And finally, PPV below 19% was observed for the three subscenarios, whereas the NPV above 82% points to the model being better at ruling out negative, than recognizing positive cases. Performance metrics are shown in Table 1.

#### Scenario II

In contrast to S1, S2 showed a much better improvement with respect to the performance metrics. Their sensitivity (49.19–52.63.19.63%) even though still modest, reflects a significantly better ability to recognize positive cases when a feature set is enriched. The CA subscenario yielded the highest sensitivity at 52.63%. Conversely, the specificity was slightly lower (64.40–87.50.40.50%) with the combination scenario achieving the highest one. The AUROC ranging from 56.97–71.46.97.46% showed a better than at random prediction ability. The highest value was obtained in the combination subscenario. The F1-scores increased substantially (53.57–65.30.57.30%), as well as the MCC values (0.13–0.30.13.30) which indicated a moderate positive predictive correlation for the combination subscenario. A considerable improvement relative to S1 was in the PPV (58.82–94.11.82.11%), although this is owed to the smaller negative class size. Performance metrics are shown in Table 1.

**Table 1:** Summarized evaluation metrics for Scenario I and Scenario II (German cohorts) compared to the US NMEDW cohort reported by Huda et al^13^.The number of positive and negative cases are denoted as (n=positive cases; negative cases).

	German cohorts						US cohort
**Metric**	Scenario I			Scenario II			NMEDW
	E85.4	E85.8	E85.4+E85.8	E85.4	E85.8	E85.4+E85.8	E85.4
	(n=76; 359)	(n=61; 374)	(n=96; 339)	(n=76; 44)	(n=61; 59)	(n=96; 24)	(n=261; 39393)
Sensitivity [%]	18.42	11.47	14.58	52.63	49.18	50.00	63.6
Specificity [%]	83.28	82.08	82.30	75.00	64.40	87.50	85.5
Accuracy [%]	71.95	72.18	67.35	60.83	56.66	57.50	85.3
AUROC [%]	46.98	51.48	47.85	68.12	56.97	71.46	80.0
F1-Score [%]	18.66	10.37	16.47	62.99	53.57	65.30	5.36
MCC [−1,1]	0.01	−0.05	−0.03	0.26	0.13	0.30	0.11
PPV [%]	18.91	9.45	18.91	78.43	58.82	94.11	2.8
NPV [%]	82.82	85.04	77.28	47.82	55.07	30.43	99.7

Performance evaluation of the RF model across German Scenario I and Scenario II cohorts and comparison with the US NMEDW dataset^13^.

**Fig. 5:**
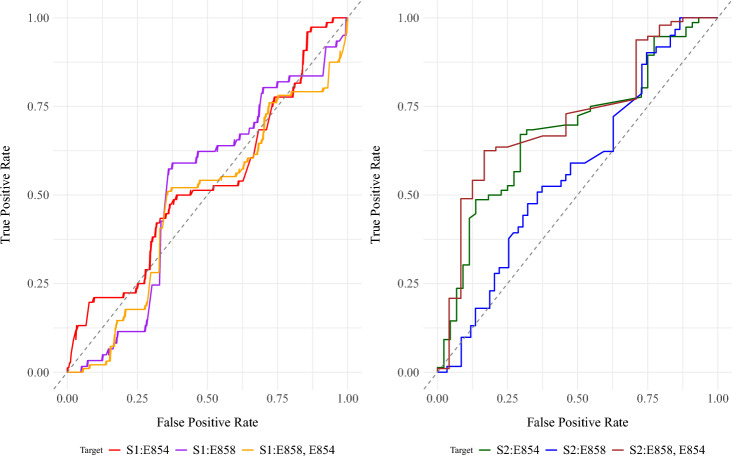
In S1 (left), AUROC values ranged from 46.98% to 51.48%, indicating near-random discrimination. In contrast, S2 (right) showed improved performance, with AUROC values ranging from 56.97% to 71.46%.

As can be seen in Fig. 5, the lowest values of AUROC were obtained in all three S1, whereas S2 performed significantly better. The highest AUROC was obtained at 71% for the combination subscenario, followed by 68.12% for the CA subscenario, and lastly, 56.97% for the ATTRwt-CM subscenario in S2. Confusion matrices can be consulted in the Supplementary Results.

Ultimately, the RF model showed potential in identifying previously undiagnosed cases, provided further analyses, refinement, and development. Bootstrapped 95% confidence intervals for all reported metrics are provided in the Supplementary Results.

## Methods

### German data sources

ATTR-CM cases were collected from an interdisciplinary amyloidosis center (ACCB) which operates digitally through its registry database TBase, a cross-campus, digital care structure for long-term research of patients with amyloidosis and currently harbors $\sim$800 patients^19^. The initial collection resulted in 181 patients, from which ATTRwt patients were selected, supported by negative genetic tests for ATTRv. Routinely collected data that can be considered as secondary and tertiary care, based on ICD-10-GM coding were obtained from the registry retrospectively, covering a time span from 2002 to 2023. This amounted to a total of 946 unique ICD-10-GM codes that were subjected to a version mapping (ICD-10 v.2019) with the R package ICD10gm (v. 1.2.5), which offers functionality to aid in the identification, specification, and historization of ICD-10-GM codes^20^. The mapping was necessary to track the potential changes in the codes’ definitions from their original year of entry to the ICD-10 version adapted in 2019.

Remaining suitable cases were sourced from the Systemic Amyloidosis Screening in Patients with Aortic Valve Stenosis (SAVER) study, comprising HF patients awaiting transcatheter (TAVR) or surgical aortic valve replacement (SAVR). As opposed to the ACCB cohort’s nature of longitudinal data, the SAVER patients’ information was gathered in the form of multiple questionnaires containing information about cardiological, neurological, and psychological symptoms, from which the appropriate ICD-10-CM codes were identified. Questionnaires captured diagnoses in a binary format (presence vs. absence), for example by asking whether a patient had been diagnosed with carpal tunnel syndrome (neurological questions) or atrial fibrillation (cardiological questions). Affirmative responses were subsequently mapped to the corresponding ICD-10-CM codes. No psychological questions were eligible for mapping to an ICD-10-CM code. The study was approved by the local ethical research committee, Ethical committee of Charité – Universitätsmedizin Berlin (reference number: EA1/014/20 and EA4/325/20). Patients and members of the public were not involved in the design, conduct, reporting, or dissemination plans of this research. All methods were performed in accordance with the relevant guidelines and regulations.

### ICD-10 code mapping and RF model validation setup

The version-mapped codes underwent a three-step process to map the ICD-10-GM to the ICD-10-CM via: (1) exact match mapping, (2) partial match mapping, and (3) manual code inspection for codes with significantly differing definitions between the two ICD-10 systems. All three subsets have been approved by cardiologists and neurologists. A RF ML model, developed by Huda et al^13^., was employed for validation on the German data set. The features of the model comprised 1872 ICD-10-CM codes, selected from medical claims data by filtering diagnoses present in at least 2% of their original US-based ATTRwt-CM cohort (300 million patients). Inspired by Huda et al.’s^13^ target variable definitions, which both reflect common coding practices for ATTRwt (i.e., E85.4 with the definition of organ-limited amyloidosis and E85.82 for ATTRwt), we follow this principle and define multiple target variables to account for differences in coding systems, while preserving the medical soundness of appropriate disease documentation. The prediction of the E85.4 target is strengthened by a prior exclusion of patients with diagnostic codes indicative of the alternative form, AL-CM amyloidosis. This ensures only ATTRwt as the actual target. Following the original author’s^13^ example, we denote this target as the CA target since it reflects a clinically informed definition. In accordance with Huda et al.’s^13^approach of additionally evaluating the more specific ATTRwt code as the target (E85.82), we assessed the performance of the RF model using a second target variable based on coding practices of ATTRwt in the German setting. For this purpose, we used the code E85.8 (defined as “other amyloidosis”), which is commonly applied by physicians in Germany to document ATTRwt due to the lack of the E85.82 code in the GM. Finally, we defined a combined target variable including both E85.4 and E85.8 to assess whether providing an enriched positive class improves the performance of the RF model. The rationale underlying the individual target definitions also applies to this combined scenario. All target definitions, as well as the original work by Huda et al^13^., were restricted to patients with HF, where each target variable was required to co-occur with a corresponding HF diagnosis code, and thereby refers specifically to ATTRwt with cardiac manifestation, denoted as ATTRwt-CM. Given that the positive cases originating from the ACCB already have a definitive ATTRwt diagnosis, the focus has been directed to evaluating if the US-based RF model will predict them correctly, as well as how the model will perform predicting on a HF cohort (SAVER), which potentially contains unrecognized ATTR-CM patients. Additionally, considering the differing sources of the data with respect to their nature, i.e. retrospectively gathered vs. questionnaire-based, led to the formation of two scenarios for the ML validation on the German cohort:

(i) Scenario 1 (S1)— Since the ACCB cohort had much more diagnosis counts aligning with its retrospective nature, a feature constraint was imposed, defined as restricting the input feature space to diagnoses that are specific for SAVER. During RF model validation, only the features present in both, SAVER and ACCB were retained in order to avoid bias introduction due to the skewed feature availability in the two datasets. All other features were considered as absent. This resulted in 54 intersecting features, as shown in Fig. 4, illustrating the overlaps between the Huda features and ACCB, Huda and SAVER, and the number of positive features allowed in S1, represented by the bar for Huda, SAVER and ACCB.
(ii) Scenario 2 (S2) corresponded to validating the RF on a data set from ACCB, without any feature constraints. This yielded 640 overlapping features between ACCB and the RF feature set as shown in Fig. 4, where their relationships to the other feature sets can be seen.

Both S1 and S2 share three subscenarios, defined based on three differing target variables. To this end and as mentioned above, we distinguish the CA subscenario with the codes E85.4 and HF as the target, the ATTRwt-CM with the codes E85.8 and HF, and the combination scenario defined as E85.4 and HF, or E85.8 and HF as the target variable. The CA scenario was also evaluated on the US-based, external and unbalanced Northwestern Medicine Enterprise Data Warehouse (NMEDW) dataset by Huda et al^13^. Therefore, the same target variable was used to directly compare the performance on the German dataset. Important to note is the fact that the ATTRwt-CM is an adjusted version of the target variable since the GM does not define the specific ATTRwt code. Nevertheless, the code E85.8 is commonly used in the clinical practice to define ATTRwt cases. Here, the evaluation focuses on how well ATTRwt-CM patients are predicted by the RF model, i.e. on the generalizability of the model. Finally, given the combination scenario we aim to evaluate the model’s performance with an enriched positive class, as well as estimate how well the RF model can predict either CA or ATTRwt-CM.

Labeling of the positive and negative classes for the RF model was based on the respective presence of the ICD-10-CM codes for CA (E85.) or the adjusted ATTRwt-CM (E85.8) as the positive class, depending on the employed scenario. The negative class cohort consists of all patients assigned ICD-10-CM codes related to HF, specified by ICD-10 codes I50.0–I50.9, covering a total of 29 potential codes and excluded amyloidosis diagnoses. For S1 (CA), the model ultimately labeled 76 ATTRwt-CM patients as positive cases (E85.4 + HF codes) and 359 HF patients as negative cases, whereas for S2 (CA) 76 were positive, and 44 patients were labeled as negative.

Huda et al.’s^13^ work also employed similar clinical scenarios, among others that were matched by propensity based on certain factors. The RF validation on the US-based NMEDW cohort closely aligns with the characteristics of the German CA scenario based not only on the class imbalance, but also on the fact that these data were not matched by propensity and correspond to an unmanipulated case of patient data.

### Data preparation and patient selection

**Fig. 6:**
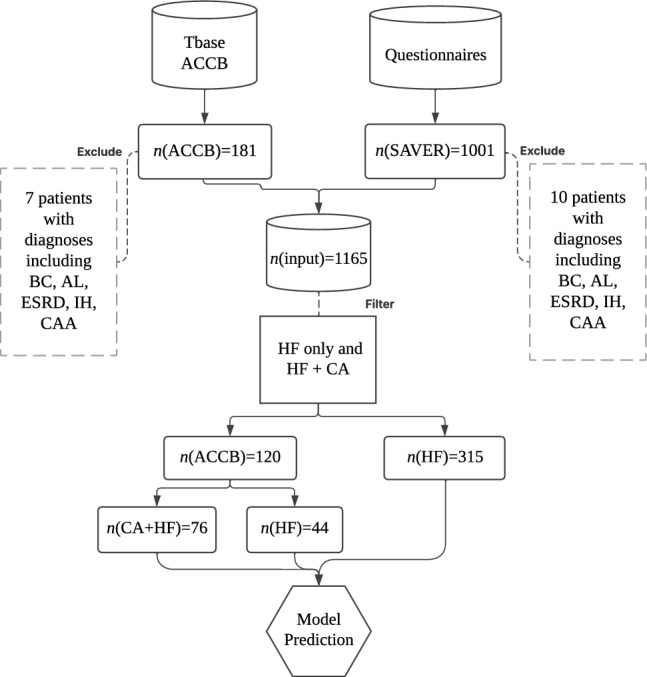
From data preparation to model prediction (CA scenario). Combining the initial patient cohorts, the ACCB (TBase), gathered retrospectively and summarizing information from questionnaires employed by SAVER resulted in 181 and 1001 patients, respectively. Respecting the exclusion constraints for 17 patients (7 from ACCB, 10 from SAVER) left 1165 patients. The ones without HF codes were left out. The remaining set was divided into patients with a combination of CA and HF as the positive class, and patients containing only HF codes as the negative class. Ultimately, 120 patients from ACCB were selected and divided based on the presence of CA and HF (n=76) codes and HF (n=44) codes. 315 patients from the SAVER study were recognized as HF. At last, the model divided the original input into 76 CA and 359 HF patients. Excluded were patients with diagnoses: blood cancer (BC), light-chain amyloidosis (AL), end stage renal disease (ESRD), and cerebral amyloid angiopathy (CAA).

Of the initial 181 ATTRwt patients collected from ACCB, 120 were retained for RF model input after preprocessing. Inclusion criteria required the presence of a HF code from the ICD-10 chapter I50 (HF), in combination with at least one amyloidosis code (E85.8 and/or E85.4). From the initial 1001 patients from the SAVER study, preprocessing resulted in 315 HF patients, based on the presence of HF codes. An overview of the process can be seen in Fig. 6.

As mentioned, S1 was composed of patients from both ACCB and SAVER and just as S2, was divided into three subscenarios based on respective scenarios’ subsets of positive cases, consisting of 76 patients in the CA subscenario (target variable E85.4), 61 patients for the ATTRwt-CM subscenario (target variable E85.8), and finally 96 patients in the combination subscenario (target variable E85.4 and/or E85.8). A feature constraint was imposed for S1 – since the SAVER-questionnaire study had much less diagnoses codes relative to the retrospectively gathered ACCB diagnoses, only the diagnoses codes present in SAVER were allowed for both patient groups in this validation setting. Finally, recognizing the potential bias in S1, S2 aimed to evaluate the model’s performance on a uniform source of data— only ACCB.

## Discussion

Evaluating a predictive RF model trained on US-based medical claims data, for identifying patients at risk for developing the rare disease ATTRwt-CM among HF patients in a German setting was comprised of multiple classification approaches with respect to the target variable to assess its robustness and clinical applicability in a single center study. Direct application of the US-algorithm to the German data set was not possible due to semantic and coding system differences. Here, only mitigation strategies were applied by the formation of two scenarios. We limited the number of features to our questionnaire-based dataset SAVER, which had significantly larger patient numbers, yet for the expense of feature richness. Here, we could evaluate how much predictive quality suffers when rolling out to wider, but less specified population. In our second scenario, we resorted to a smaller patient group, but with a significantly larger feature overlap to the originally employed features of Huda et al^13^. This can serve as a best-case scenario, yet there are still semantic differences in the utilization of even correctly mapped codes. Here, we can see the potential gains of using an internationally unified and semantically standardized patient characterization.

Due to the absence of specific codes for ATTRwt in the ICD-10-GM, and the inconsistent use of related codes (E85.4 or E85.8), defining a reliable negative class was challenging. This highlights the broader issue of ambigous coding practices, which are intensified when comparing US and German data sets. While the CM and GM define the code E85.4 equally as “organ-limited amyloidosis”, the uncertainty of geographically specific usages remain at question. This influences documentation and affects the recognition, as well as identification of ATTR-CM in routine clinical data.

As to be expected, Huda et al.’s^13^ RF model has demonstrated moderate predictive power for the feature enriched S2, but had significantly lower prediction power for the feature constrained S1, almost down to random guessing. This further highlights the adherence to internationally standardized patient documentation in routine care, ensuring a larger number of semantically identical features in the future. Though the model showed potential, it is still far from being implemented into clinical systems to offer support for clinicians. Despite the demonstrated transferability of the feature sets through the modular mapping approach (GM to CM), as evidenced by the AUROC values in S2, semantically harmonized data that extend across borders for AI-supported risk identification, particularly in the context of rare diseases have yet to be established. Specific limitations with respect to cohort selection must be addressed as well. The selection of positive ATTR-CM cases from an already known source that contains ATTRwt-CM patients (ACCB register), as well as the selection of a HF group which shows a predisposition for ATTRwt-CM is a bias for the assessment of a screening model performance. An unbiased approach would include screening for patients at risk in a random cohort of HF patients. However, given that the RF relies on the ICD-10-CM, validation outside US would likely result in feature loss, discrepancies in feature definitions and ambiguities arising from differing and uncertain coding practices. The features that are not defined in a specific coding system, but are a part of the RF feature set, are marked as absent, which is misleading and does not reflect on the true state of the patient’s health. Additionally, utilizing medical claims data for following disease trajectories poses certain challenges. They have historically primarily served as administrative or billing purposes, rather than detailed clinical documentation. This may limit their representativeness for research applications and must be considered.

Huda et al’s^13^ RF model was an opportunity for our first out of two steps for inspecting the possibilities of shortening the path to rare disease diagnosis in the German setting. The former step of inspecting the single-center routine data of the ACCB serves as the first building block on our way to evaluating the multi-center states of data integration centers of German university hospitals, in which a semantically harmonized setting will serve as the basis for gaining knowledge about symptom profiles of ATTR-CM. Depending on the outcomes, the results will be used to evaluate the deductive power of the profiles in identifying previously unsuspected ATTR-CM cases in German data integration centers. This is planned as a part of the Collaboration for Rare Disease Observation and Research (LABRADOR-for-ATTRwt), set within the Screen4Care framework.

## Conclusion

Our findings demonstrate that cross-border transfer of ICD-10-based ML models for rare disease identification is strongly limited, even under enriched conditions such as those in our single-center study design. Because our evaluation was based on a preselected, biased cohort rather than a true population-wide screening, the observed model performance cannot be directly extrapolated to broader clinical settings. Nevertheless, these results highlight the inherent limitations of relying solely on routine coding systems and suggest that greater semantic harmonization, as well as the integration of rare-disease-focused ontologies such as HPO^17^ and ORPHAcodes^16^, could improve the generalizability and clinical utility of ML-assisted rare disease detection.

## Supplementary Information

Supplementary Information.

## Data Availability

Data is available at reasonable request.

## Code availability

The code used for the analysis, including the mapping approaches is not publicly available, since it is not generalizable to external datasets or institutions. It has been tailored to the specific structure of data at hand.
