# Supplementary material for: Transferability of a US claims-based machine learning model for ATTRwt-CM identification: a retrospective evaluation in a German setting
Source: Sci Rep. 2026 Apr 24;16:18966. doi: 10.1038/s41598-026-49636-3 (PMC13275804; doi:10.1038/s41598-026-49636-3)
Supplement: Supplementary file 1 — Supplementary Information. [file 41598_2026_49636_MOESM1_ESM.pdf]

Supplementary Information

Transferability of a US claims-based machine learning model for ATTRwt-CM identification: a retrospective evaluation in a German setting

Harisa Muratovic-Colic[1,2,9], Miriam R. Hübner[3], Jan-Filip Rehburg[3], Isabel Mattig[1,3,4,5,6], Paul J. Wetzel[1,2], Katrin Wrede-Wihl[1,5], Helena F. Pernice[1,2,3], Gina Barzen[1,4,5,6], Nicolas Wieder[1,2,3], Jakub Piwowarski[1,4,6], Stephan Bohl[8], Vera von Landenberg-Roberg[8], Daniel Messroghli[1,7], Sebastian Spethmann[1,4,5,6], Richard Röttger[9], Josef Schepers[1,3,†], Katrin Hahn[1,2,3,†]

[†]These authors contributed equally to this work.

[1]Amyloidosis Center Charité Berlin (ACCB), Charité Universitätsmedizin Berlin, Berlin, Germany
[2]Klinik für Neurologie mit Experimenteller Neurologie, Charité Universitätsmedizin Berlin, Berlin, Germany
[3]Berlin Institute of Health (BIH) at Charité, Charité Universitätsmedizin Berlin, Berlin, Germany
[4]Charité – Universitätsmedizin Berlin, corporate member of Freie Universität Berlin and Humboldt-Universität zu Berlin, Charitéplatz 1, 10117, Berlin, Germany
[5]DZHK (German Centre for Cardiovascular Research), Partner Site Berlin, Charité Universitätsmedizin Berlin, Berlin, Germany
[6]Department of Cardiology, Angiology and Intensive Care Medicine, Campus Charité Mitte, Deutsches Herzzentrum der Charité (DHZC), Charitéplatz 1, 10117, Berlin, Germany
[7]Department of Cardiology, Rhythmology and Angiology, Medical University of Lausitz – Carl Thiem, Cottbus, Germany
[8]Medizinische Klinik mit Schwerpunkt Hämatologie, Onkologie und Tumorimmunologie, Charité Universitätsmedizin Berlin, Berlin, Germany
[9]Department of Mathematics and Computer Science, University of Southern Denmark, Odense, Denmark

Supplementary Methods

ICD-10-GM to ICD-10-CM Mapping - ACCB

The mapping approach was initiated by scaling the 946 ICD-10-GM codes from the ACCB to the same ICD-10 version. As Huda et al.[1] used both the 2016 and 2019 versions, we used the 2019 version to map the GM codes to the same version with the R package ICD10gm (v.1.2.5.)[2]. Since differences between the 2019 and 2023 GM versions were minimal and mainly affected COVID-19-related additions, the 2019-standardized codes were considered equivalent to the 2023 GM version. This allowed us to directly map to the ICD-10-CM 2023 version, obtained from the National Center for Health Statistics via the CDC[3] website (CDC ICD-10-CM April 2023 Update).
The CM codes and definitions were stored within the archive "icd10cm-Order-CodeFiles2023.zip", from which we used the file "icd10cm-order-2023.txt" (please note that the file naming conventions have since been updated in the archive to "icd10cm-code descriptions- April 1 2023.zip", while the content of the Fiscal Year 2023 remains unchanged).

As mentioned, we employed a modular mapping approach, such that we divided our GM codes into three groups – exact matches, partial matches, and manually inspected codes to map them accordingly to the CM. Starting with the 946 GM codes gathered from the ACCB, in the approach named exact match mapping, we denoted the GM dataset as G and the CM dataset as C, and we merge G and C by the codes that align exactly. This was done for 721 codes. For the remaining 225 codes, a partial matching approach was applied by aligning codes based on their first three ICD-10 characters in both G and C. A total of 119 codes fit this partial mapping approach. The remaining 106 codes, which could not be aligned through sequence-based matching, were manually reviewed by identifying the closest corresponding definitions within the ICD-10-CM system (2023). These codes, as well as the partial matches were discussed and approved by physicians to ensure the medical soundness of the mapping. Definition examples can be consulted in Figure 2 in the main text, and an overview of the methodology in the Algorithm 1 below. Given the differences in the coding systems and the mapping approaches, we obtained a total of 870 mapped codes to the CM.

Algorithm 1 ICD-10-GM to ICD-10-CM Mapping

Input:

G = set of ICD-10-GM codes (ACCB cohort) ▷ 946 codes

C = set of ICD-10-CM codes (`icd10cm-order-2023.txt`)

Step 0: Consensus ICD-10-GM version

Convert all codes in G to ICD-10-GM version 2019

Step 1: Exact Match Mapping

$E \leftarrow \{g \in G \mid g$ exactly matches a code in $C\}$ ▷ 721 codes

$R_1 \leftarrow G \setminus E$ ▷ 225 remaining unmatched GM codes

Step 2: Partial Match Mapping (3-character level)

for each code $r \in R_1$ **do**

    if first 3 characters of r match any code in C **then**

        Assign r to corresponding CM code

    end if

end for

$P \leftarrow$ set of partially matched codes ▷ 119 codes

$R_2 \leftarrow R_1 \setminus P$ ▷ 106 remaining unmatched codes

Step 3: Manual Mapping

for each code $r \in R_2$ **do**

    Identify closest matching definition in C

    Assign corresponding CM code based on clinical judgment

end for

$M \leftarrow$ set of manually mapped codes

Step 4: Validation

Review P and M with physicians to ensure clinical consistency

Output:

Final mapped set = $E \cup P \cup M$ ▷ 870 mapped codes

ICD-10-GM to ICD-10-CM Mapping - SAVER

The SAVER questions assessing the presence or absence of neurological and cardiological diagnoses such as “carpal tunnel syndrome” or “essential hypertension” were manually looked up in the `icd10cm-order-2023.txt` file. All codes were reviewed by physicians to ensure appropriateness.

Supplementary Results

Table 1: Confusion matrices for Scenario I (left) and Scenario II (right).

Scenario I — **T1: E85.4+HF**

		Predicted	
		Positive	Negative
Actual	Positive	14	62
	Negative	60	299

Scenario II — **T1: E85.4+HF**

	Predicted	
	Positive	Negative
Positive	40	36
Negative	11	33

Scenario I — **T2: E85.8+HF**

		Predicted	
		Positive	Negative
Actual	Positive	7	54
	Negative	67	307

Scenario II — **T2: E85.8+HF**

	Predicted	
	Positive	Negative
Positive	30	31
Negative	21	38

Scenario I — **T3: E85.4+E85.8+HF**

		Predicted	
		Positive	Negative
Actual	Positive	14	82
	Negative	60	279

Scenario II — **T3: E85.4+E85.8+HF**

	Predicted	
	Positive	Negative
Positive	40	36
Negative	11	33

To assess the stability of the reported performance metrics, we computed bootstrapped 95% confidence intervals (CIs) for the respective scenarios (1000 iterations, sampling with replacement). Analysis was performed in Python (v.3.12.1) with scikit-learn (v.1.7.2)[4].

Table 2: Bootstrapped performance metrics, Scenario I. Values are presented as mean [95% CI].

	**Scenario I**		
**Metric**	**E85.4 + HF**	**E85.8 + HF**	**E85.8 + E85.4+ HF**
Specificity	0.833 [0.794–0.870]	0.821 [0.780–0.856]	0.823 [0.782–0.863]
Recall	0.186 [0.103–0.273]	0.116 [0.036–0.201]	0.147 [0.080–0.219]
Accuracy	0.720 [0.678–0.761]	0.722 [0.680–0.761]	0.674 [0.629–0.715]
AUROC	0.471 [0.399–0.542]	0.516 [0.439–0.589]	0.479 [0.414–0.543]
F1-score	0.187 [0.104–0.270]	0.104 [0.031–0.176]	0.165 [0.094–0.241]
MCC	0.019 [-0.073–0.113]	-0.058 [-0.143–0.031]	-0.032 [-0.118–0.062]
PPV	0.191 [0.107–0.284]	0.096 [0.028–0.169]	0.191 [0.107–0.284]
NPV	0.829 [0.792–0.868]	0.851 [0.814–0.885]	0.773 [0.731–0.816]

Table 3: Bootstrapped performance metrics, Scenario II. Values are presented as mean [95% CI].

	**Scenario II**		
**Metric**	**E85.4 + HF**	**E85.8 + HF**	**E85.8 + E85.4 + HF**
Specificity	0.749 [0.622–0.870]	0.646 [0.522–0.771]	0.873 [0.731–1.000]
Recall	0.526 [0.412–0.644]	0.492 [0.375–0.614]	0.500 [0.396–0.602]
Accuracy	0.608 [0.517–0.700]	0.567 [0.483–0.658]	0.575 [0.492–0.667]
AUROC	0.679 [0.575–0.777]	0.572 [0.471–0.677]	0.712 [0.592–0.816]
F1-score	0.627 [0.525–0.722]	0.534 [0.420–0.641]	0.651 [0.550–0.740]
MCC	0.268 [0.096–0.427]	0.139 [-0.023–0.320]	0.301 [0.163–0.434]
PPV	0.782 [0.673–0.889]	0.590 [0.455–0.730]	0.940 [0.870–1.000]
NPV	0.479 [0.362–0.603]	0.551 [0.440–0.667]	0.306 [0.203–0.421]

References

[1] Huda, A., Castaño, A., Niyogi, A., et al. *A machine learning model for identifying patients at risk for wild-type transthyretin amyloid cardiomyopathy.* Nature Communications. 2021;12:2725. https://doi.org/10.1038/s41467-021-22876-9

[2] Donnachie, E. *ICD10gm: Metadata Processing for the German Modification of the ICD-10 Coding System.* 2023. Available at: https://edonnachie.github.io/ICD10gm/ https://doi.org/10.5281/zenodo.2542833

[3] National Center for Health Statistics, Classification of Diseases, Functioning, and Disability. *ICD-10-CM April 2023 Update.* Centers for Disease Control and Prevention. 2023. Available at: https://ftp.cdc.gov/pub/Health_Statistics/NCHS/Publications/ICD10CM/April-1-2023-Update/ [Accessed: 02.04.2026]

[4] Pedregosa, F., Varoquaux, G., Gramfort, A., et al. *Scikit-learn: Machine Learning in Python.* Journal of Machine Learning Research. 2011;12:2825–2830.
